# Crystal structure of (*E*)-3-(4-hy­droxy­benz­yl)-4-{[4-(methyl­sulfan­yl)benzyl­idene]amino}-1*H*-1,2,4-triazole-5(4*H*)-thione

**DOI:** 10.1107/S2056989015021994

**Published:** 2015-11-21

**Authors:** P. S. Manjula, B. K. Sarojini, B. Narayana, K. Byrappa, S. Madan Kumar

**Affiliations:** a Department of Chemistry, PA College of Engineering, Nadupadavu 574 153, D.K., Mangaluru, India; b Department of Industrial Chemistry, Mangalagangotri, Mangalore University, Mangaluru 574 199, India; c Department of Chemistry, Mangalagangotri, Mangalore University, Mangaluru 574 199, India; d Department of Materials Science, Mangalagangotri, Mangalore University, Mangaluru 574 199, India; e PURSE Lab, Mangalagangotri, Mangalore University, Mangaluru 574 199, India

**Keywords:** crystal structure, triazole, thione, methyl­thio­benzyl­idene, hydrogen bonding, π–π inter­actions

## Abstract

In the title compound, C_17_H_16_N_4_OS_2_, the triazole and methyl­thio­benzyl­idene rings are nearly coplanar, making a dihedral angle of 6.52 (12)°. An intra­molecular C—H⋯S hydrogen bond forms an *S*(6) ring motif. The hy­droxy­benzyl ring is almost normal to the triazole and methyl­thio­benzyl­idene rings, making dihedral angles of 78.56 (12) and 84.79 (11)°, respectively. In the crystal, mol­ecules are linked through O—H⋯N and N—H⋯O hydrogen bonds, forming layers parallel to the *ac* plane. The layers are linked *via* C—H⋯N hydrogen bonds, forming a three-dimensional structure. In addition, a short π–π inter­action is observed [inter-centroid distance = 3.764 (3) Å], involving inversion-related methyl­thio­benzyl­idene rings.

## Related literature   

For the structure of a related compound, see: Manjula *et al.* (2015 ▸).
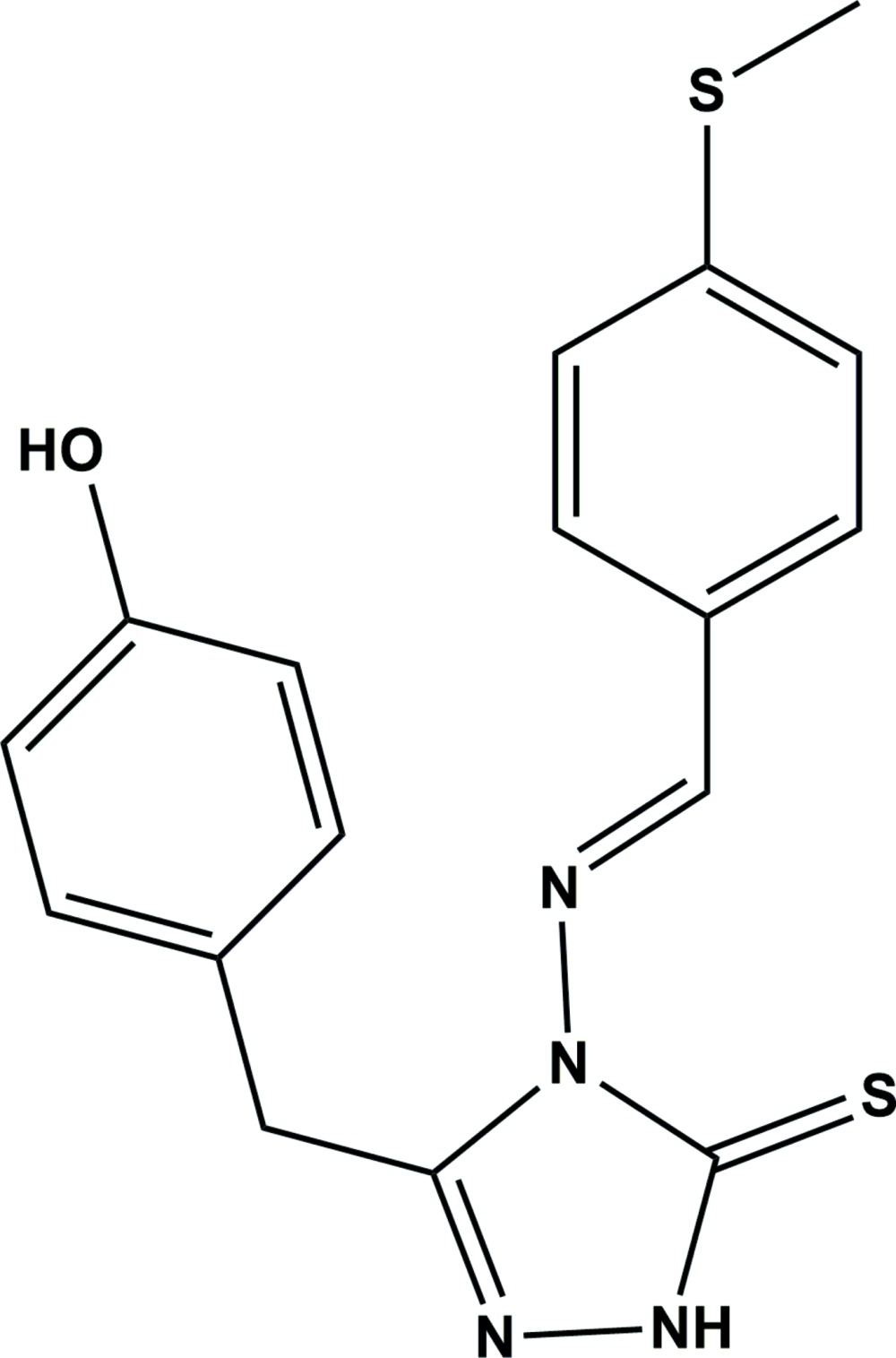


## Experimental   

### Crystal data  

C_17_H_16_N_4_OS_2_
*M* *_r_* = 356.46
Monoclinic, 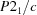

*a* = 7.739 (5) Å
*b* = 28.161 (16) Å
*c* = 7.945 (4) Å
β = 100.407 (11)°
*V* = 1703.0 (17) Å^3^
*Z* = 4
Mo *K*α radiation
μ = 0.32 mm^−1^
*T* = 293 K
0.57 × 0.34 × 0.24 mm

### Data collection  

Rigaku Saturn724+ diffractometer
Absorption correction: numerical (*NUMABS*; Rigaku, 1999 ▸) *T*_min_ = 0.895, *T*_max_ = 0.954
8210 measured reflections
3017 independent reflections
2200 reflections with *I* > 2σ(*I*)
*R*_int_ = 0.031

### Refinement  

*R*[*F* ^2^ > 2σ(*F* ^2^)] = 0.047
*wR*(*F* ^2^) = 0.107
*S* = 1.06
3017 reflections
217 parameters
H-atom parameters constrained
Δρ_max_ = 0.16 e Å^−3^
Δρ_min_ = −0.17 e Å^−3^

### 

Data collection: *CrystalClear* (Rigaku, 2011 ▸); cell refinement: *CrystalClear*; data reduction: *CrystalClear*; program(s) used to solve structure: *SHELXS97* (Sheldrick, 2008 ▸); program(s) used to refine structure: *SHELXL2014* (Sheldrick, 2015 ▸); molecular graphics: *Mercury* (Macrae *et al.*, 2008 ▸); software used to prepare material for publication: *OLEX2* (Dolomanov *et al.*, 2009 ▸).

## Supplementary Material

Crystal structure: contains datablock(s) I. DOI: 10.1107/S2056989015021994/su5236sup1.cif

Structure factors: contains datablock(s) I. DOI: 10.1107/S2056989015021994/su5236Isup2.hkl

Supporting information file. DOI: 10.1107/S2056989015021994/su5236Isup3.cml

**Fig. 1.**
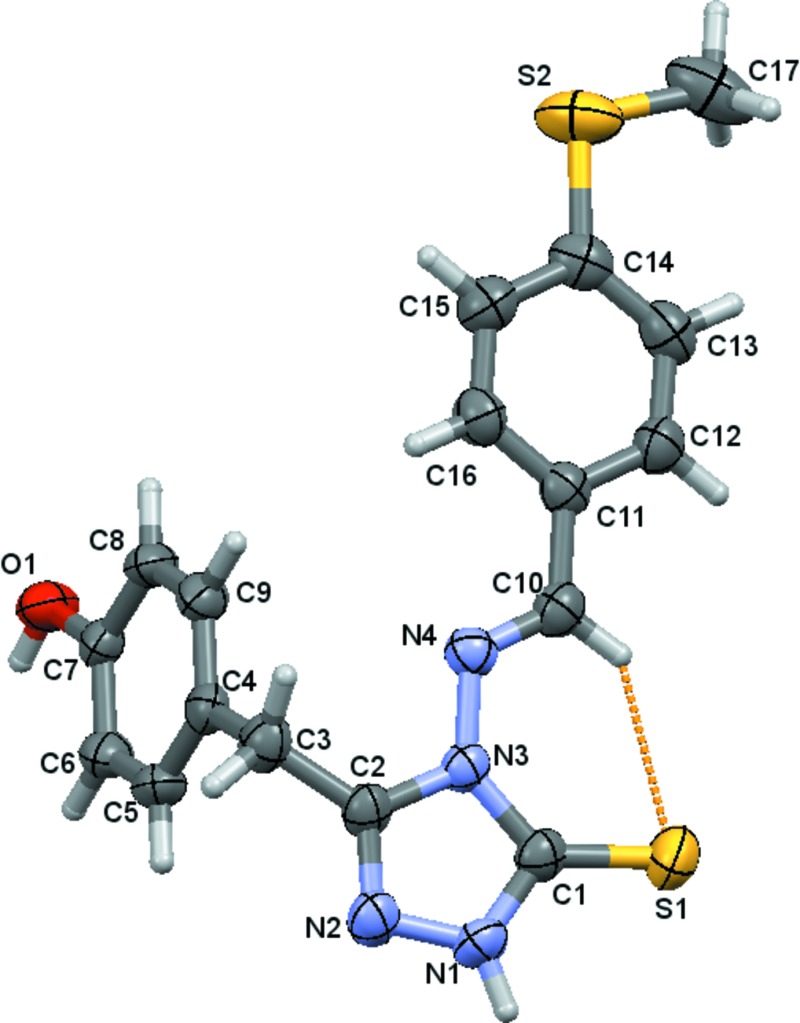
A view of the molecular structure of the title compound, with atom labelling. Displacement ellipsoids are drawn at the 50% probability level and the intramolecular C—H···S hydrogen bond is drawn as a dashed line (see Table 1).

**Fig. 2.**
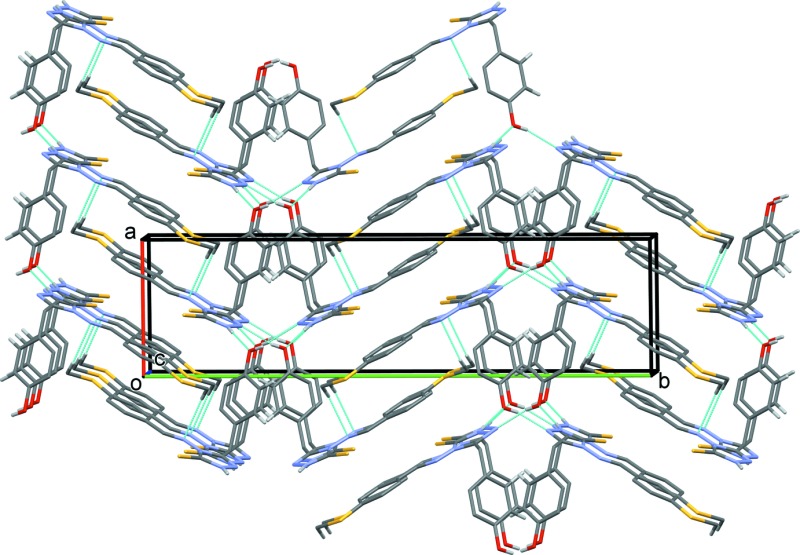
A viewed along the c axis of the crystal packing of the title compound. Hydrogen bonds are drawn as a dashed lines (see Table 1), and H atoms not involved in hydrogen bonding have been omitted for clarity.

. DOI: 10.1107/S2056989015021994/su5236fig1.tif
A view of the mol­ecular structure of the title compound, with atom labelling. Displacement ellipsoids are drawn at the 50% probability level and the intra­molecular C—H⋯S hydrogen bond is drawn as a dashed line (see Table 1).

c . DOI: 10.1107/S2056989015021994/su5236fig2.tif
A viewed along the *c* axis of the crystal packing of the title compound. Hydrogen bonds are drawn as a dashed lines (see Table 1), and H atoms not involved in hydrogen bonding have been omitted for clarity.

**Fig. 3.**
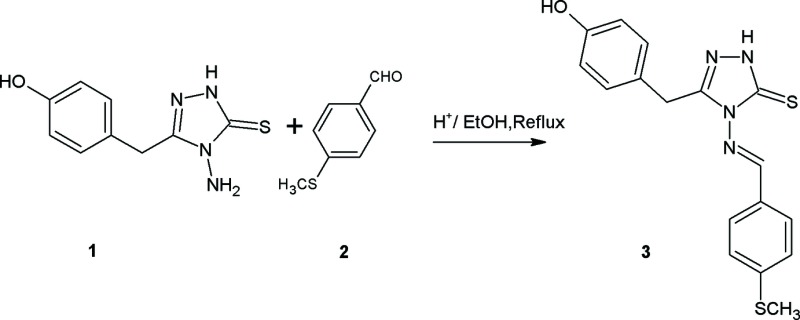
Reaction scheme.

. DOI: 10.1107/S2056989015021994/su5236fig3.tif
Reaction scheme.

CCDC reference: 1437595

Additional supporting information:  crystallographic information; 3D view; checkCIF report

## Figures and Tables

**Table 1:** Hydrogen-bond geometry (Å, °)

*D*—H⋯*A*	*D*—H	H⋯*A*	*D*⋯*A*	*D*—H⋯*A*
C10—H10⋯S1	0.93	2.52	3.267 (3)	138
O1—H1⋯N2^i^	0.82	2.03	2.806 (3)	159
N1—H1*A*⋯O1^ii^	0.86	1.98	2.816 (3)	164
C17—H17*C*⋯N4^iii^	0.96	2.62	3.472 (4)	148

Symmetry codes: (i) 
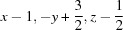
; (ii) 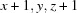; (iii) 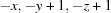.

## supplementary crystallographic information

### S1. Comment

The title compound was synthesized, crystallized and its crystal structure is presented as part of our work on 3-methyl-1*H*-1,2,4-triazole-5(4*H*)-thione derivatives (Manjula *et al.*, 2015).

The molecular structure of the title compound is as shown in Fig 1. The methylsulfanylbenzylidene and triazole rings are almost coplanar with a dihedral angle of 6.52 (12) °. The hydroxybenzyl ring makes dihedral angles of 78.56 (12) ° and 84.79 (11) ° with the triazole and methylthiobenzylidene rings, respectively. An intramolecular interaction of the type C10—H10···S1 is observed (Fig. 1 and Table 1).

In the crystal, the molecules are connected through O1—H1···N2 and N1—H1A···O1 hydrogen bonds (Table 1) forming layers parallel to (010). The layers are linked by C17—H17C···N4 hydrogen bonds forming a three-dimensional structure (Fig. 2 and Table 1) . In addition, a parallel slipped π–π (Cg···Cg^i^) interaction is observed with an inter-centroid distance of 3.764 (3) Å [Cg is the centroid of ring C11—C16; inter-planar distance = 3.500 (1) Å; slippage 1.384 Å; symmetry code: (i) -x, -y+1, -z+1].

### S2. Experimental

The synthesis of title compound, (3), is illustrated in Fig. 3. A suspension of 4-(methylthio)benzaldehyde (2) (0.01 mol) in ethanol (15 ml) was added to 4-amino-3-(4-hydroxybenzyl)-1*H*-1,2,4-triazole-5(4*H*)-thione (1) (0.01 mol) and heated until a clear solution was obtained. To this a few drops of conc. H_2_SO_4_ were added as a catalyst and the mixture was refluxed for 36 h on a water bath. The precipitate formed was filtered and recrystallized from methanol to give the titled compound. Single crystals were obtained by recrystallization from acetic acid (m.p. 469—471 K).

### S3. Refinement

Crystal data, data collection and structure refinement details are summarized in Table 2. The H atoms were fixed geometrically (O-H = 0.82 Å, N-H = 0.86 Å, and C—H = 0.93–0.97 Å) and allowed to ride on their parent atoms with *U*_iso_(H) = 1.2*U*_eq_(O,N,C).

### Crystal data

**Table:**

C_17_H_16_N_4_OS_2_	*F*(000) = 744
*M**_r_* = 356.46	*D*_x_ = 1.390 Mg m^−^^3^
Monoclinic, *P*2_1_/*c*	Mo *K*α radiation, λ = 0.71075 Å
*a* = 7.739 (5) Å	Cell parameters from 2021 reflections
*b* = 28.161 (16) Å	θ = 3.0–25.3°
*c* = 7.945 (4) Å	µ = 0.32 mm^−^^1^
β = 100.407 (11)°	*T* = 293 K
*V* = 1703.0 (17) Å^3^	Prism, yellow
*Z* = 4	0.57 × 0.34 × 0.24 mm

### Data collection

**Table:**

Rigaku Saturn724+ diffractometer	2200 reflections with *I* > 2σ(*I*)
Detector resolution: 7.111 pixels mm^-1^	*R*_int_ = 0.031
profile data from ω–scans	θ_max_ = 25.3°, θ_min_ = 3.0°
Absorption correction: numerical (*NUMABS*; Rigaku, 1999)	*h* = −9→9
*T*_min_ = 0.895, *T*_max_ = 0.954	*k* = −33→33
8210 measured reflections	*l* = −9→6
3017 independent reflections	

### Refinement

**Table:**

Refinement on *F*^2^	Primary atom site location: structure-invariant direct methods
Least-squares matrix: full	Hydrogen site location: inferred from neighbouring sites
*R*[*F*^2^ > 2σ(*F*^2^)] = 0.047	H-atom parameters constrained
*wR*(*F*^2^) = 0.107	*w* = 1/[σ^2^(*F*_o_^2^) + (0.0424*P*)^2^ + 0.239*P*] where *P* = (*F*_o_^2^ + 2*F*_c_^2^)/3
*S* = 1.06	(Δ/σ)_max_ < 0.001
3017 reflections	Δρ_max_ = 0.16 e Å^−^^3^
217 parameters	Δρ_min_ = −0.17 e Å^−^^3^
0 restraints	

### Special details

**Table:**

Geometry. All e.s.d.'s (except the e.s.d. in the dihedral angle between two l.s. planes) are estimated using the full covariance matrix. The cell e.s.d.'s are taken into account individually in the estimation of e.s.d.'s in distances, angles and torsion angles; correlations between e.s.d.'s in cell parameters are only used when they are defined by crystal symmetry. An approximate (isotropic) treatment of cell e.s.d.'s is used for estimating e.s.d.'s involving l.s. planes.

### Fractional atomic coordinates and isotropic or equivalent isotropic displacement parameters (Å^2^)

**Table:**

	*x*	*y*	*z*	*U*_iso_*/*U*_eq_	
S1	0.53353 (10)	0.58782 (3)	1.04260 (8)	0.0612 (2)	
S2	0.00577 (11)	0.40779 (3)	0.16264 (10)	0.0797 (3)	
O1	−0.2083 (2)	0.71862 (6)	0.21145 (19)	0.0526 (5)	
H1	−0.2399	0.7431	0.2531	0.063*	
N1	0.6203 (2)	0.67074 (7)	0.9170 (2)	0.0482 (5)	
H1A	0.6603	0.6818	1.0173	0.058*	
N2	0.6227 (3)	0.69644 (7)	0.7707 (2)	0.0469 (5)	
N3	0.5039 (2)	0.62570 (6)	0.7141 (2)	0.0386 (5)	
N4	0.4256 (2)	0.59134 (7)	0.5984 (2)	0.0410 (5)	
C1	0.5514 (3)	0.62736 (9)	0.8925 (3)	0.0414 (6)	
C2	0.5495 (3)	0.66794 (8)	0.6491 (3)	0.0403 (6)	
C3	0.5120 (3)	0.68017 (9)	0.4640 (3)	0.0490 (6)	
H3A	0.5476	0.6539	0.3991	0.059*	
H3B	0.5815	0.7077	0.4448	0.059*	
C4	0.3195 (3)	0.69085 (8)	0.3991 (3)	0.0401 (6)	
C5	0.2392 (3)	0.72987 (8)	0.4562 (3)	0.0482 (6)	
H5	0.3045	0.7500	0.5360	0.058*	
C6	0.0633 (3)	0.73960 (8)	0.3971 (3)	0.0465 (6)	
H6	0.0115	0.7661	0.4371	0.056*	
C7	−0.0348 (3)	0.70997 (8)	0.2788 (3)	0.0407 (6)	
C8	0.0426 (3)	0.67050 (8)	0.2227 (3)	0.0494 (6)	
H8	−0.0234	0.6499	0.1447	0.059*	
C9	0.2177 (3)	0.66139 (8)	0.2818 (3)	0.0470 (6)	
H9	0.2689	0.6348	0.2418	0.056*	
C10	0.3768 (3)	0.55258 (9)	0.6531 (3)	0.0459 (6)	
H10	0.3949	0.5468	0.7702	0.055*	
C11	0.2918 (3)	0.51667 (8)	0.5332 (3)	0.0414 (6)	
C12	0.2277 (3)	0.47597 (9)	0.5961 (3)	0.0497 (6)	
H12	0.2421	0.4717	0.7138	0.060*	
C13	0.1424 (3)	0.44141 (9)	0.4884 (4)	0.0539 (7)	
H13	0.1007	0.4142	0.5337	0.065*	
C14	0.1194 (3)	0.44748 (9)	0.3131 (3)	0.0497 (6)	
C15	0.1865 (3)	0.48812 (9)	0.2491 (3)	0.0524 (7)	
H15	0.1737	0.4923	0.1314	0.063*	
C16	0.2715 (3)	0.52218 (9)	0.3575 (3)	0.0477 (6)	
H16	0.3157	0.5491	0.3125	0.057*	
C17	−0.0520 (4)	0.35993 (11)	0.2856 (4)	0.0858 (10)	
H17A	−0.1153	0.3365	0.2109	0.103*	
H17B	0.0526	0.3459	0.3497	0.103*	
H17C	−0.1246	0.3714	0.3629	0.103*	

### Atomic displacement parameters (Å^2^)

**Table:**

	*U*^11^	*U*^22^	*U*^33^	*U*^12^	*U*^13^	*U*^23^
S1	0.0752 (5)	0.0629 (5)	0.0440 (4)	0.0047 (4)	0.0069 (3)	0.0112 (3)
S2	0.0756 (6)	0.0803 (6)	0.0843 (6)	−0.0262 (5)	0.0171 (4)	−0.0331 (5)
O1	0.0503 (11)	0.0521 (11)	0.0503 (10)	0.0091 (9)	−0.0044 (8)	−0.0126 (8)
N1	0.0483 (13)	0.0511 (13)	0.0395 (12)	−0.0014 (10)	−0.0079 (9)	−0.0028 (10)
N2	0.0412 (12)	0.0476 (12)	0.0472 (12)	−0.0023 (10)	−0.0044 (9)	0.0051 (10)
N3	0.0346 (11)	0.0411 (11)	0.0378 (11)	0.0003 (9)	0.0006 (9)	0.0015 (9)
N4	0.0386 (11)	0.0393 (12)	0.0436 (11)	−0.0004 (9)	0.0037 (9)	−0.0020 (9)
C1	0.0350 (13)	0.0458 (14)	0.0408 (14)	0.0068 (11)	0.0003 (11)	0.0002 (11)
C2	0.0316 (13)	0.0440 (14)	0.0429 (14)	0.0006 (11)	0.0003 (11)	0.0046 (11)
C3	0.0462 (15)	0.0543 (16)	0.0460 (15)	−0.0058 (13)	0.0069 (12)	0.0074 (12)
C4	0.0475 (14)	0.0401 (14)	0.0321 (13)	−0.0017 (11)	0.0050 (11)	0.0062 (10)
C5	0.0586 (17)	0.0409 (14)	0.0401 (14)	−0.0040 (13)	−0.0043 (12)	−0.0070 (11)
C6	0.0572 (17)	0.0395 (14)	0.0408 (14)	0.0062 (12)	0.0031 (12)	−0.0076 (11)
C7	0.0479 (15)	0.0406 (14)	0.0310 (12)	0.0054 (12)	−0.0003 (11)	0.0018 (10)
C8	0.0614 (17)	0.0404 (14)	0.0394 (14)	0.0050 (13)	−0.0095 (12)	−0.0086 (11)
C9	0.0564 (17)	0.0400 (14)	0.0418 (14)	0.0114 (12)	0.0014 (12)	−0.0044 (11)
C10	0.0437 (15)	0.0490 (16)	0.0435 (14)	0.0027 (12)	0.0036 (11)	0.0025 (12)
C11	0.0338 (13)	0.0399 (14)	0.0492 (15)	0.0039 (11)	0.0044 (11)	0.0015 (11)
C12	0.0501 (15)	0.0494 (16)	0.0467 (15)	0.0022 (13)	0.0013 (12)	0.0054 (12)
C13	0.0489 (16)	0.0422 (15)	0.0712 (19)	−0.0012 (13)	0.0121 (14)	0.0045 (13)
C14	0.0373 (14)	0.0497 (16)	0.0635 (18)	0.0009 (12)	0.0126 (12)	−0.0084 (13)
C15	0.0505 (16)	0.0605 (17)	0.0456 (15)	0.0015 (14)	0.0071 (12)	−0.0031 (13)
C16	0.0473 (15)	0.0461 (15)	0.0511 (16)	−0.0039 (12)	0.0125 (12)	0.0036 (12)
C17	0.063 (2)	0.0600 (19)	0.139 (3)	−0.0127 (16)	0.031 (2)	−0.029 (2)

### Geometric parameters (Å, º)

**Table:**

S1—C1	1.655 (2)	C6—H6	0.9300
S2—C14	1.752 (3)	C6—C7	1.377 (3)
S2—C17	1.769 (3)	C7—C8	1.375 (3)
O1—H1	0.8200	C8—H8	0.9300
O1—C7	1.374 (3)	C8—C9	1.376 (3)
N1—H1A	0.8600	C9—H9	0.9300
N1—N2	1.372 (2)	C10—H10	0.9300
N1—C1	1.333 (3)	C10—C11	1.462 (3)
N2—C2	1.304 (3)	C11—C12	1.378 (3)
N3—N4	1.396 (2)	C11—C16	1.386 (3)
N3—C1	1.399 (3)	C12—H12	0.9300
N3—C2	1.368 (3)	C12—C13	1.382 (3)
N4—C10	1.258 (3)	C13—H13	0.9300
C2—C3	1.487 (3)	C13—C14	1.383 (3)
C3—H3A	0.9700	C14—C15	1.390 (3)
C3—H3B	0.9700	C15—H15	0.9300
C3—C4	1.517 (3)	C15—C16	1.375 (3)
C4—C5	1.379 (3)	C16—H16	0.9300
C4—C9	1.383 (3)	C17—H17A	0.9600
C5—H5	0.9300	C17—H17B	0.9600
C5—C6	1.385 (3)	C17—H17C	0.9600
			
C14—S2—C17	104.78 (14)	C7—C8—H8	120.0
C7—O1—H1	109.5	C7—C8—C9	120.0 (2)
N2—N1—H1A	122.4	C9—C8—H8	120.0
C1—N1—H1A	122.4	C4—C9—H9	119.2
C1—N1—N2	115.23 (18)	C8—C9—C4	121.6 (2)
C2—N2—N1	103.43 (19)	C8—C9—H9	119.2
N4—N3—C1	133.95 (19)	N4—C10—H10	119.9
C2—N3—N4	117.69 (18)	N4—C10—C11	120.2 (2)
C2—N3—C1	108.36 (19)	C11—C10—H10	119.9
C10—N4—N3	119.70 (19)	C12—C11—C10	119.3 (2)
N1—C1—S1	126.50 (18)	C12—C11—C16	118.4 (2)
N1—C1—N3	101.73 (19)	C16—C11—C10	122.3 (2)
N3—C1—S1	131.78 (19)	C11—C12—H12	119.2
N2—C2—N3	111.2 (2)	C11—C12—C13	121.6 (2)
N2—C2—C3	124.8 (2)	C13—C12—H12	119.2
N3—C2—C3	123.9 (2)	C12—C13—H13	120.1
C2—C3—H3A	109.0	C12—C13—C14	119.8 (2)
C2—C3—H3B	109.0	C14—C13—H13	120.1
C2—C3—C4	112.74 (18)	C13—C14—S2	124.5 (2)
H3A—C3—H3B	107.8	C13—C14—C15	118.8 (2)
C4—C3—H3A	109.0	C15—C14—S2	116.8 (2)
C4—C3—H3B	109.0	C14—C15—H15	119.5
C5—C4—C3	121.3 (2)	C16—C15—C14	120.9 (2)
C5—C4—C9	117.7 (2)	C16—C15—H15	119.5
C9—C4—C3	121.0 (2)	C11—C16—H16	119.8
C4—C5—H5	119.3	C15—C16—C11	120.5 (2)
C4—C5—C6	121.3 (2)	C15—C16—H16	119.8
C6—C5—H5	119.3	S2—C17—H17A	109.5
C5—C6—H6	120.1	S2—C17—H17B	109.5
C7—C6—C5	119.9 (2)	S2—C17—H17C	109.5
C7—C6—H6	120.1	H17A—C17—H17B	109.5
O1—C7—C6	122.5 (2)	H17A—C17—H17C	109.5
O1—C7—C8	118.0 (2)	H17B—C17—H17C	109.5
C8—C7—C6	119.5 (2)		
			
S2—C14—C15—C16	177.44 (19)	C2—C3—C4—C5	65.8 (3)
O1—C7—C8—C9	177.6 (2)	C2—C3—C4—C9	−113.4 (2)
N1—N2—C2—N3	−0.7 (2)	C3—C4—C5—C6	−179.9 (2)
N1—N2—C2—C3	176.7 (2)	C3—C4—C9—C8	179.6 (2)
N2—N1—C1—S1	178.79 (16)	C4—C5—C6—C7	0.0 (3)
N2—N1—C1—N3	−0.8 (2)	C5—C4—C9—C8	0.3 (3)
N2—C2—C3—C4	−103.6 (3)	C5—C6—C7—O1	−177.9 (2)
N3—N4—C10—C11	178.90 (18)	C5—C6—C7—C8	1.0 (3)
N3—C2—C3—C4	73.4 (3)	C6—C7—C8—C9	−1.4 (3)
N4—N3—C1—S1	0.2 (4)	C7—C8—C9—C4	0.7 (4)
N4—N3—C1—N1	179.7 (2)	C9—C4—C5—C6	−0.7 (3)
N4—N3—C2—N2	−179.30 (18)	C10—C11—C12—C13	178.4 (2)
N4—N3—C2—C3	3.4 (3)	C10—C11—C16—C15	−178.2 (2)
N4—C10—C11—C12	−175.2 (2)	C11—C12—C13—C14	−0.3 (4)
N4—C10—C11—C16	4.2 (3)	C12—C11—C16—C15	1.1 (3)
C1—N1—N2—C2	0.9 (3)	C12—C13—C14—S2	−177.14 (19)
C1—N3—N4—C10	2.8 (3)	C12—C13—C14—C15	1.4 (4)
C1—N3—C2—N2	0.2 (3)	C13—C14—C15—C16	−1.2 (4)
C1—N3—C2—C3	−177.1 (2)	C14—C15—C16—C11	−0.1 (4)
C2—N3—N4—C10	−177.9 (2)	C16—C11—C12—C13	−1.0 (4)
C2—N3—C1—S1	−179.21 (18)	C17—S2—C14—C13	−3.9 (2)
C2—N3—C1—N1	0.3 (2)	C17—S2—C14—C15	177.60 (19)

### Hydrogen-bond geometry (Å, º)

**Table:**

*D*—H···*A*	*D*—H	H···*A*	*D*···*A*	*D*—H···*A*
C10—H10···S1	0.93	2.52	3.267 (3)	138
O1—H1···N2^i^	0.82	2.03	2.806 (3)	159
N1—H1*A*···O1^ii^	0.86	1.98	2.816 (3)	164
C17—H17*C*···N4^iii^	0.96	2.62	3.472 (4)	148

Symmetry codes: (i) *x*−1, −*y*+3/2, *z*−1/2; (ii) *x*+1, *y*, *z*+1; (iii) −*x*, −*y*+1, −*z*+1.
