# Supplementary figures and images for: Crystal structure of (E)-3-(4-hy­droxy­benz­yl)-4-{[4-(methyl­sulfan­yl)benzyl­idene]amino}-1H-1,2,4-triazole-5(4H)-thione
Source: Acta Crystallogr E Crystallogr Commun. 2015 Nov 21;71(Pt 12):o982–3. doi: 10.1107/S2056989015021994 (PMC4719935; doi:10.1107/S2056989015021994)

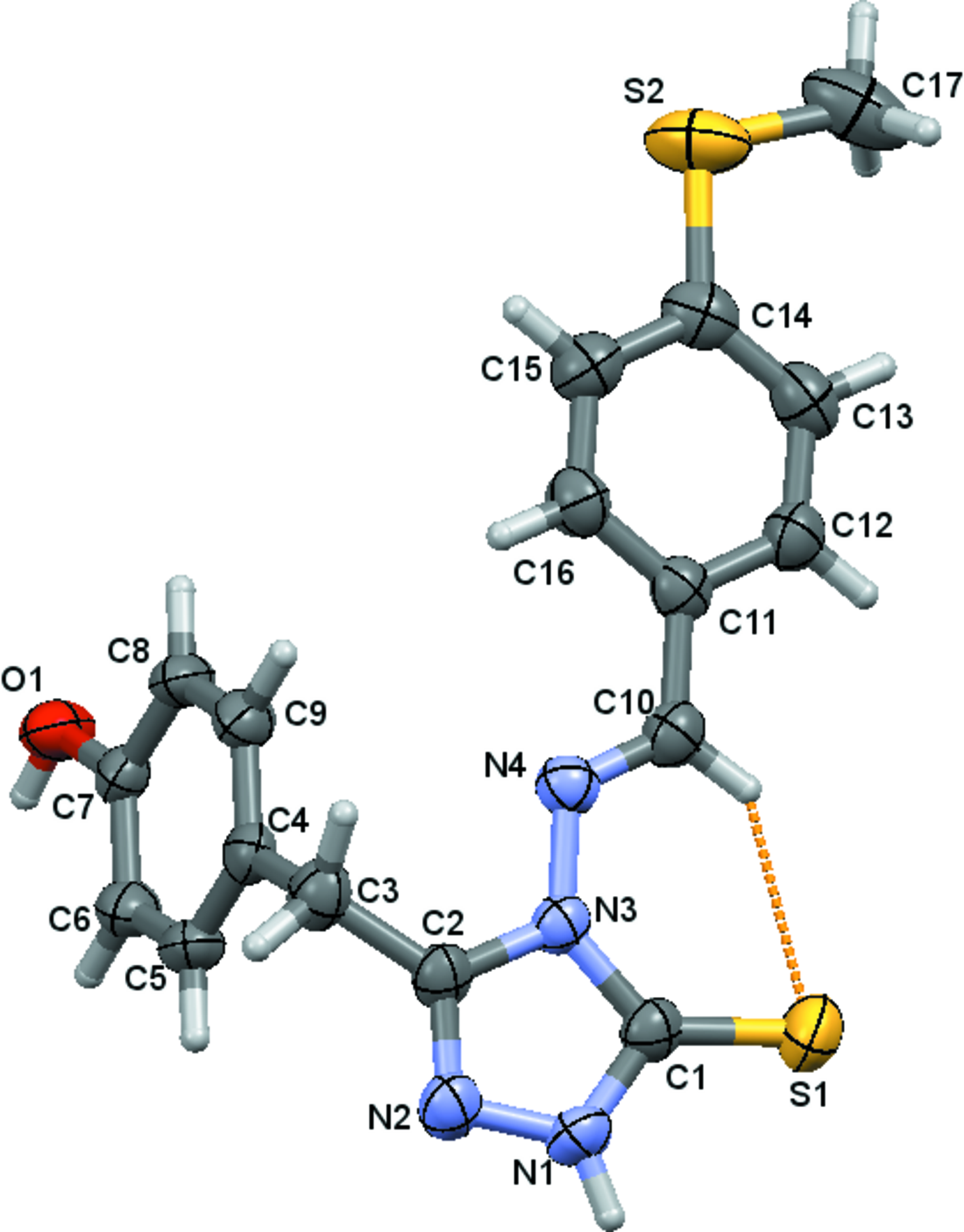

Supplement: Supplementary file 4 [file e-71-0o982-fig1.tif]

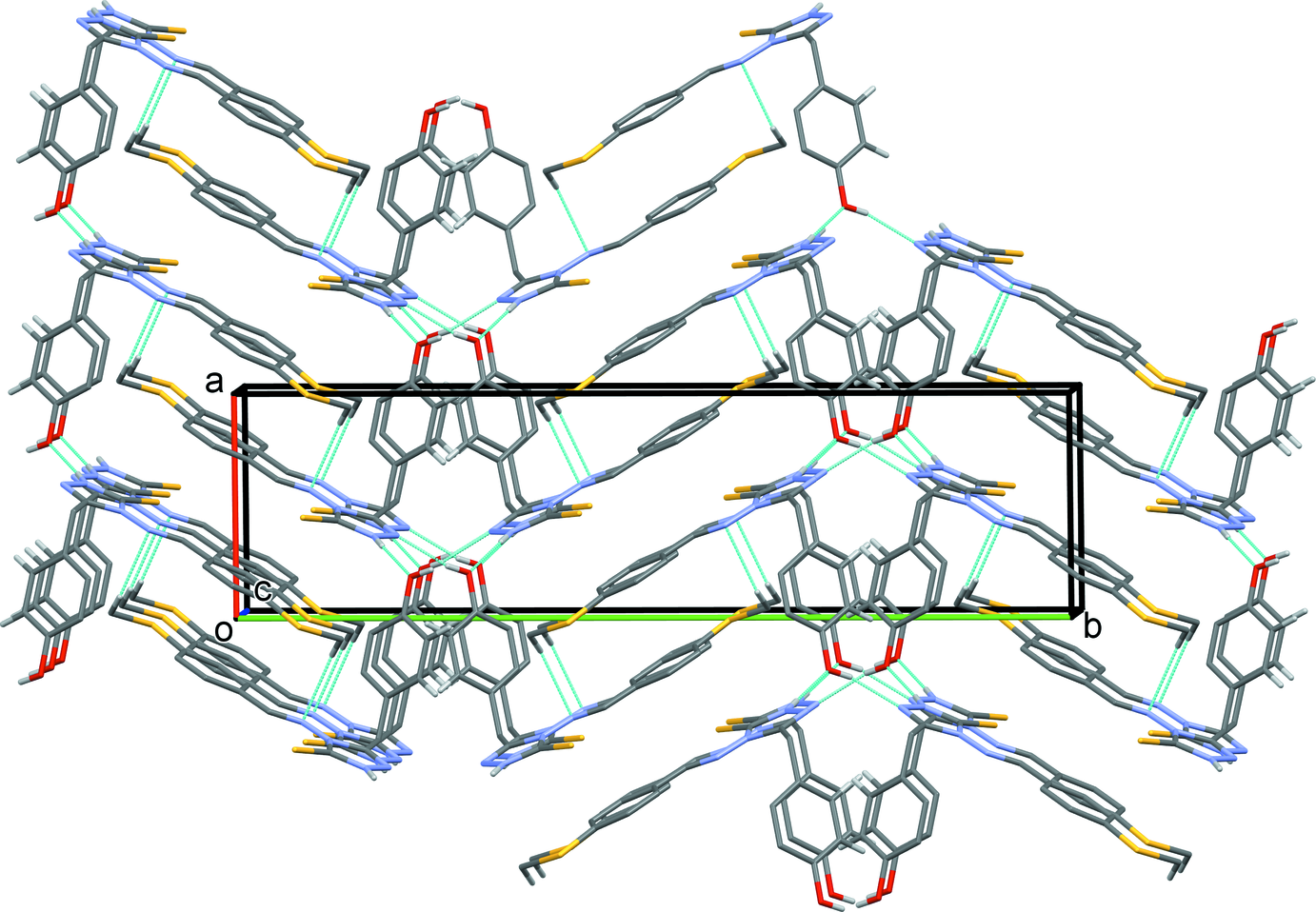

Supplement: Supplementary file 5 [file e-71-0o982-fig2.tif]

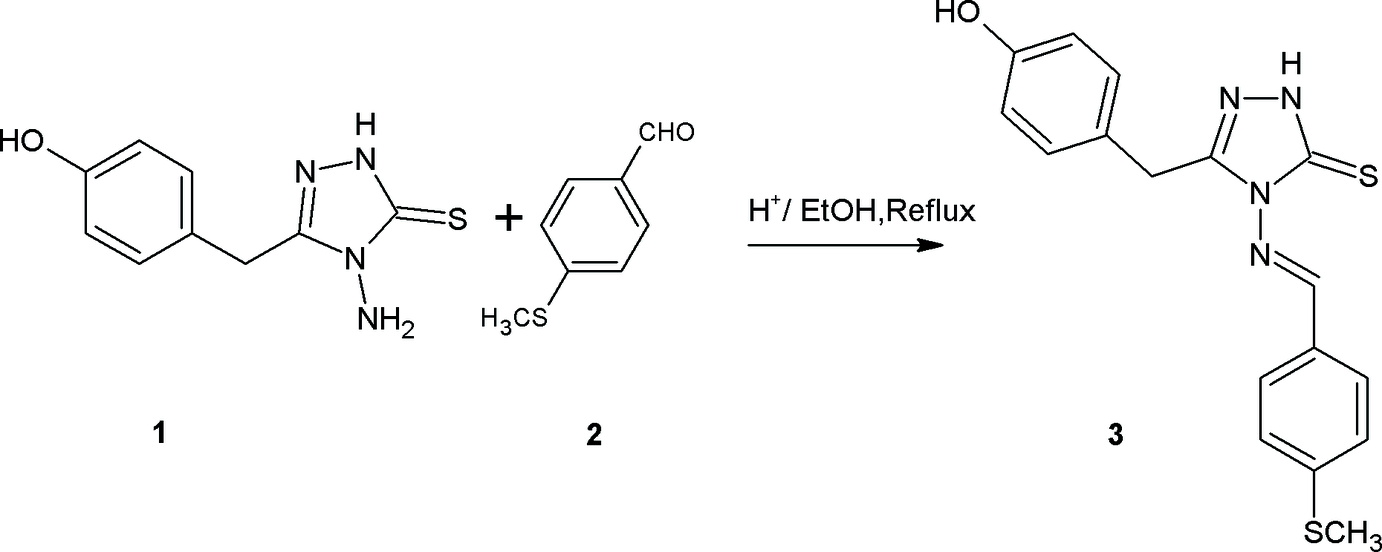

Supplement: Supplementary file 6 [file e-71-0o982-fig3.tif]
